# LncRNA CYTOR affects the proliferation, cell cycle and apoptosis of hepatocellular carcinoma cells by regulating the miR-125b-5p/KIAA1522 axis

**DOI:** 10.18632/aging.202306

**Published:** 2020-12-09

**Authors:** Bo Hu, Xiao-Bo Yang, Xu Yang, Xin-Ting Sang

**Affiliations:** 1 Department of Liver Surgery, Peking Union Medical College Hospital, Chinese Academy of Medical Sciences and Peking Union Medical College, Beijing 100730, China

**Keywords:** lncRNA CYTOR, hepatocellular carcinoma cells, proliferation, apoptosis, miR-125b-5p/KIAA1522 axis

## Abstract

We aimed to investigate whether lncRNA CYTOR could sponge miR-125b-5p to affect hepatocellular carcinoma (HCC) cells through targeting KIAA1522. The expression of CYTOR, miR-125b-5p and KIAA1522 in HCC cells was detected by Real-time quantitative polymerase chain reaction (RT-qPCR) analysis. KIAA1522 expression in HCC tissues was detected by immunohistochemistry. The proliferation, cell cycle and apoptosis of HCC cells after transfection were respectively detected by Cell Counting Kit-8 (CCK-8) assay and flow cytometry analysis, and related protein expression was determined by Western blot analysis. As a result, The Cancer Genome Atlas (TCGA) database indicated that expression of CYTOR and KIAA1522 was increased in HCC tissues and high expression of CYTOR and KIAA1522 was related to worse overall survival. MiR-125b-5p expression was decreased in HCC tissues, which was negatively correlated with the expression of CYTOR and KIAA1522. The proliferation and cell cycle of HCC cells were suppressed by CYTOR interference while promoted by miR-125b-5p interference and KIAA1522 overexpression. The apoptosis of HCC cells was promoted by CYTOR interference while inhibited by miR-125b-5p interference and KIAA1522 overexpression. In conclusion, CYTOR interference suppressed the proliferation and cell cycle, and promoted the apoptosis of HCC cells by regulating the miR-125b-5p/KIAA1522 axis.

## INTRODUCTION

Global cancer statistics show that there are about 841,000 new cases and 782,000 deaths from liver cancer every year. Among all malignancies, liver cancer ranks sixth in incidence and fourth in mortality [1, 2]. Hepatocellular carcinoma (HCC) accounts for 75-85% of primary liver cancer [1], and more than 50% of HCC cases occurs in China [3]. As most HCC patients are diagnosed in the middle or advanced stage, and effective treatment methods and anti-tumor drugs are lacking, the prognosis of HCC patients is not optimistic, with the 5-year survival rate less than 40% [4]. Therefore, it is of great clinical significance to search for biomarkers used for the diagnosis and treatment of HCC.

Long non-coding RNAs (lncRNAs) are a class of non-coding RNAs with more than 200 base sequences and no protein-coding potentiality [5]. LncRNA CYTOR was up-regulated in glioma cells and tissues, and CYTOR overexpression partially reversed the inhibition of UPF1 on the proliferation and migration of glioma [6]. CYTOR was highly expressed in colorectal cancer and is associated with prognosis. CYTOR/miR-3679-5p/MACC1 axis played an important role in tumorigenesis [7]. CYTOR sponges miR-195 to promote the proliferation, migration and invasion of non-small cell lung cancer cells, and to induce cell resistance to radiation [8]. The targeted miRNAs of CYTOR predicted by starBase 3.0 and down-regulated miRNAs in HCC were intersected to obtain miR-125b-5p. Studies showed that miR-125b-5p played the role as tumor suppressor gene and inhibited the proliferation, migration and invasion of multiple cancer cells [9–11]. The targeted mRNAs of miR-125b-5p predicted by six databases (PITA, miRmap, microT, miRanda, PicTar and TargetScan) and up-regulated mRNAs in HCC were intersected to obtain KIAA1522 and PODXL. Furthermore, KIAA1522 was found to be significantly correlated with survival time of HCC patients. Liu et al. found that the overall survival of non-small-cell lung cancer (NSCLC) patients with KIAA1522 overexpression was shorter than that of NSCLC patients with low expression of KIAA1522 [12]. In esophageal squamous cell carcinoma (ESCC), KIAA1522 overexpression promoted the malignant cell proliferation and anoikis resistance of ESCC cells [13]. MiR-125b-5p expression was decreased in breast cancer cell lines and miR-125b-5p overexpression led to declined cell proliferation, colony formation ability, cell migration and invasion through targeting KIAA1522 [9].

Therefore, the purpose of this study was to investigate the role of CYTOR in HCC, and to explore whether it could sponge miR-125b-5p to affect HCC cells through targeting KIAA1522.

## RESULTS

### CYTOR expression is increased in HCC cells and tissues

**Figure 1:**
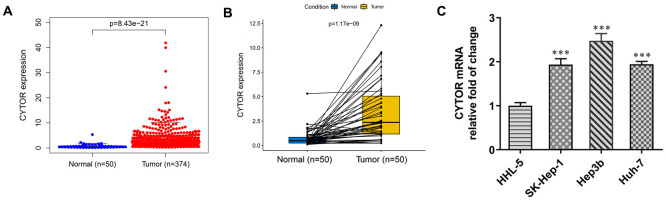
**CYTOR expression in HCC tissues and cells.** (**A**) CYTOR expression was increased in HCC tissues compared with normal tissues. (**B**) CYTOR expression was increased in HCC tissues compared with adjacent tissues. (**C**) CYTOR expression in HCC cells and HHL-5 cells was analyzed by RT-qPCR analysis. ***P<0.001 vs. HHL-5 group.

From TCGA database, CYTOR expression was increased in HCC tissues compared with normal tissues (Figure 1A) and CYTOR expression was also increased in HCC tissues compared with adjacent tissues (Figure 1B). As shown in Figure 1C, CYTOR expression was increased in HCC cells compared with HHL-5 cells. Among all HCC cells used for this study, the expression of CYTOR in Hep3b cells was the highest, and thus Hep3b cells were selected for subsequent experiments.

### CYTOR expression is related to the clinical pathological characteristics and overall survival

**Figure 2:**
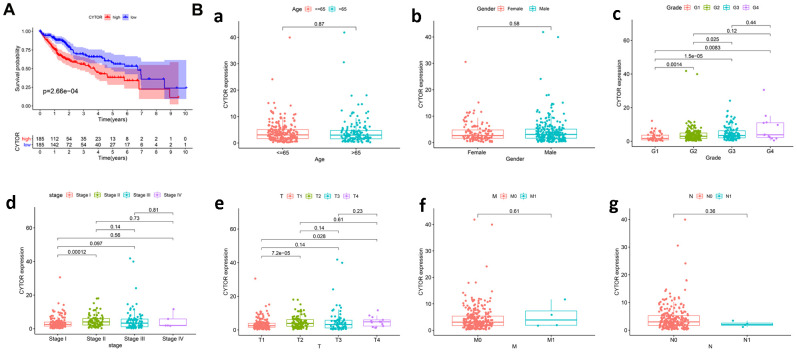
**CYTOR expression is related to the clinical pathological characteristics and overall survival.** (**A**) Impact of CYTOR expression on overall survival in HCC patients in TCGA cohort. (**B**) Association with CYTOR expression and clinicopathologic characteristics, including **a**: Age, **b**: Gender, **c**: Grade, **d**: Stage, **e**: T classification, **f**: M classification, **g**: N classification.

Kaplan-Meier curves showed that high CYTOR expression was associated with worse overall survival (Figure 2A). Differences in CYTOR expression were observed according to age, gender, grade, stage, T classification, M classification and N classification (Figure 2B). The results indicated that CYTOR expression was the highest in stage IV of HCC, and CYTOR expression was gradually elevated from G1 to G4. The associations identified between CYTOR expression and the clinical characteristics of HCC patients were shown in Table 1. Stage (P=8.07×10^-7^), T classification (P=4.73×10^-7^) and M classification (P=0.023) were significantly correlated with CYTOR expression.

**Table 1:** Univariate and multivariate analyses of correlation between CYTOR expression and overall survival in HCC patients.

**Parameter**	**Univariate analysis**				**Multivariate analysis**	
	**HR**	**95% CI**	**p-Value**	**HR**	**95% CI**	**p-Value**
Age	1.01	0.99-1.02	0.59	1.00	0.99-1.02	0.67
Gender	0.78	0.49-1.25	0.30	0.97	0.58-1.62	0.90
Grade	1.02	0.75-1.39	0.91	1.08	0.77-1.50	0.66
Stage	1.86	1.46-2.39	8.07×10^-7^	1.01	0.38-2.70	0.98
T	1.80	1.43-2.27	4.73×10^-7^	1.76	0.73-4.26	0.21
M	3.85	1.21-12.28	0.023	1.09	0.29-4.10	0.90
N	2.02	0.49-8.28	0.33	2.40	0.39-14.88	0.34
CYTOR	1.06	1.02-1.10	0.0014	1.05	1.01-1.09	0.022

HR, hazard ratio; CI, confidence interval.

### CYTOR directly targets miR-125b-5p

**Figure 3:**
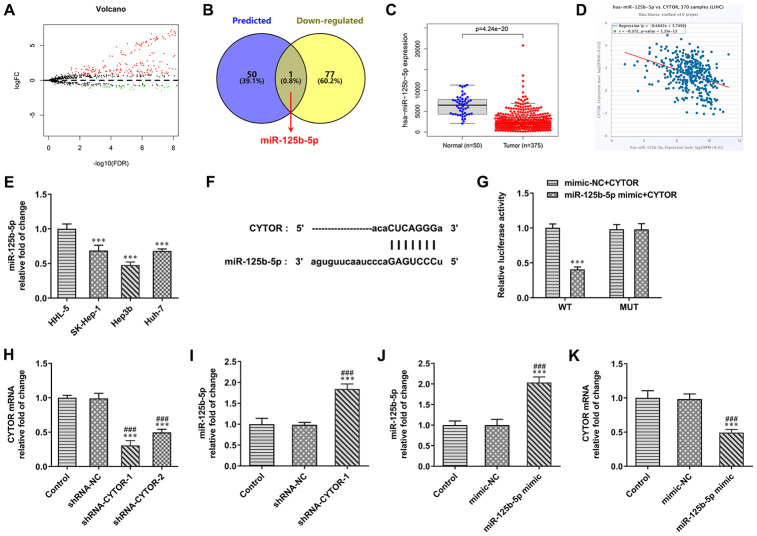
**CYTOR directly targets miR-125b-5p.** (**A**) The differentially expressed miRNAs in HCC were predicted by TCGA database. (**B**) The intersection of miRNAs combined with CYTOR was shown by Venn diagram. (**C**) miR-125b-5p expression was decreased in HCC tissues compared with normal tissues. (**D**) CYTOR expression was negatively correlated with the miR-125b-5p expression. (**E**) miR-125b-5p expression in HCC cells and HHL-5 cells was analyzed by RT-qPCR analysis. ***P<0.001 vs. HHL-5 group. (**F**) The binding site of CYTOR and miR-125b-5p was predicted by ENCORI. (**G**) Luciferase reporter assay for the confirmation of direct binding relationship between CYTOR and miR-125b-5p. ***P<0.001 vs. mimic-NC+CYTOR group. (**H**) CYTOR expression in Hep3b cells after shRNA transfection was analyzed by RT-qPCR analysis. ***P<0.001 vs. Control group. ^###^P<0.001 vs. shRNA-NC group. (**I**) miR-125b-5p expression in Hep3b cells after shRNA transfection was analyzed by RT-qPCR analysis. ***P<0.001 vs. Control group. ^###^P<0.001 vs. shRNA-NC group. (**J**) miR-125b-5p expression in Hep3b cells after mimic transfection was analyzed by RT-qPCR analysis. ***P<0.001 vs. Control group. ^###^P<0.001 vs. mimic-NC group. (**K**) CYTOR expression in Hep3b cells after mimic transfection was analyzed by RT-qPCR analysis. ***P<0.001 vs. Control group. ^###^P<0.001 vs. mimic-NC group.

The differentially expressed miRNAs in HCC were predicted by TCGA database (logFC=0.8) (Figure 3A). The Starbase 3.0 was used to predict the miRNAs that combined with CYTOR. The intersection of miRNAs that combined with CYTOR and miRNAs that were decreased in HCC was miR-125b-5p (Figure 3B). From TCGA database, miR-125b-5p expression was decreased in HCC tissues compared with normal tissues (Figure 3C). Cox regression analysis indicated that CYTOR expression was negatively correlated with the miR-125b-5p expression (Figure 3D). As shown in Figure 3E, miR-125b-5p expression was decreased in HCC cells compared with HHL-5 cells. The expression of miR-125b-5p in Hep3b cells was the lowest among all HCC cells, therefore Hep3b cells were selected for subsequent experiments. The binding site of CYTOR and miR-125b-5p were shown in Figure 3F. As shown in Figure 3G, the luciferase activity in the miR-125b-5p mimic + CYTOR WT group was decreased compared with the mimic-NC + CYTOR WT group. The luciferase activity in the miR-125b-5p mimic + CYTOR MUT group was not significantly changed compared with the mimic-NC + CYTOR MUT group. After Hep3b cells were transfected with shRNA-NC, shRNA-CYTOR-1 and shRNA-CYTOR-2, CYTOR expression was obviously decreased in the shRNA-CYTOR-1 and shRNA-CYTOR-2 groups compared with the control group and shRNA-NC group. The CYTOR expression in the shRNA-CYTOR-1 group was lower than that in the shRNA-CYTOR-2 group, and shRNA-CYTOR-1 was chosen for the next experiments (Figure 3H). After Hep3b cells were transfected with shRNA-NC and shRNA-CYTOR-1, miR-125b-5p expression was increased in Hep3b cells compared with control group and shRNA-NC group (Figure 3I). After Hep3b cells were transfected with mimic-NC and miR-125b-5p mimic, miR-125b-5p expression was increased and CYTOR expression was decreased in Hep3b cells compared with the control group and mimic-NC group (Figure 3J, 3K).

### MiR-125b-5p directly targets KIAA1522

**Figure 4:**
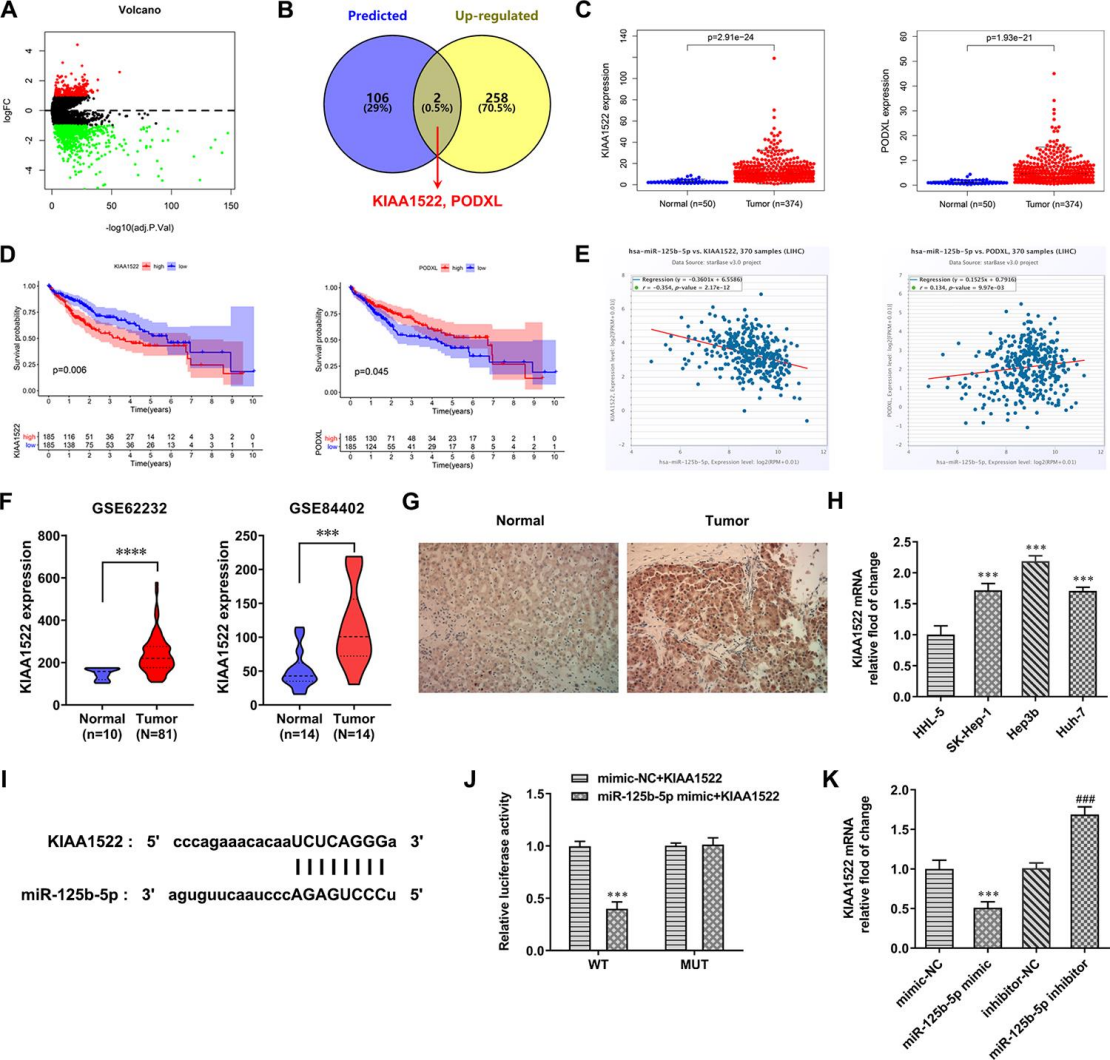
**MiR-125b-5p directly targets KIAA1522.** (**A**) The differentially expressed mRNAs in HCC were predicted by TCGA database. (**B**) The intersection of mRNAs combined with miR-125b-5p was shown by Venn diagram. (**C**) Expression of KIAA1522 and PODXL was increased in HCC tissues compared with normal tissues. (**D**) Impact of expression of KIAA1522 and PODXL on overall survival in HCC patients in TCGA cohort. (**E**) miR-125b-5p expression was negatively correlated with KIAA1522 expression. (**F**) KIAA1522 expression was increased in HCC tissues compared with normal tissues. (**G**) KIAA1522 expression in HCC tissues and adjacent tissues was detected by immunohistochemistry. (**H**) KIAA1522 mRNA expression in HCC cells and HHL-5 cells was analyzed by RT-qPCR analysis. ***P<0.001 vs. HHL-5 group. (**I**) The binding site between miR-125b-5p and KIAA1522 was predicted by ENCORI. (**J**) Luciferase reporter assay for the confirmation of direct binding relationship between miR-125b-5p and KIAA1522. ***P<0.001 vs. mimic-NC+KIAA1522 group. (**K**) KIAA1522 mRNA expression in Hep3b cells after mimic or inhibitor transfection was analyzed by RT-qPCR analysis. ***P<0.001 vs. mimic-NC group. ^###^P<0.001 vs. inhibitor-NC group.

TCGA database predicted the differentially expressed mRNAs in HCC (logFC=1) (Figure 4A). Six databases (PITA, miRmap, microT, miRanda, PicTar and TargetScan) predicted the mRNAs which were combined with miR-125b-5p. The mRNAs that not only combined with miR-125b-5p but also exhibited up-regulated expression in HCC were KIAA1522 and PODXL (Figure 4B). TCGA database predicted that the expression of KIAA1522 and PODXL in HCC tissues was increased compared with normal tissues (Figure 4C). As shown in Figure 4D, Kaplan-Meier curves showed that high KIAA1522 expression was associated with less survival probability while there was no proportional relationship of PODXL expression and survival probability. Cox regression analysis indicated that miR-125b-5p expression was negatively correlated with KIAA1522 expression (Figure 4E). Therefore, KIAA1522 was chosen for the next study. From GEO database (GSE62232 and GSE84402), KIAA1522 expression was increased in HCC tissues compared with normal tissues (Figure 4F). As shown in Figure 4G, the result from immunohistochemistry showed that KIAA1522 expression was increased in HCC tissues compared with adjacent tissues. The KIAA1522 expression was increased in HCC cells compared with HHL-5 cells. The KIAA1522 expression in Hep3b cells was the highest in HCC cells, and Hep3b cells were thus used for the next study (Figure 4H). The binding site between miR-125b-5p and KIAA1522 was shown in Figure 4I. The luciferase activity was decreased in the miR-125b-5p mimic + KIAA1522 WT group while not changed in the other three groups (Figure 4J). After Hep3b cells were transfected with mimic-NC, miR-125b-5p mimic, inhibitor-NC and miR-125b-5p inhibitor, KIAA1522 expression was increased in the miR-125b-5p mimic group compared with the mimic-NC group while decreased in the miR-125b-5p inhibitor group compared with the inhibitor-NC group (Figure 4K).

### High KIAA1522 expression is associated with cell cycle

**Figure 5:**
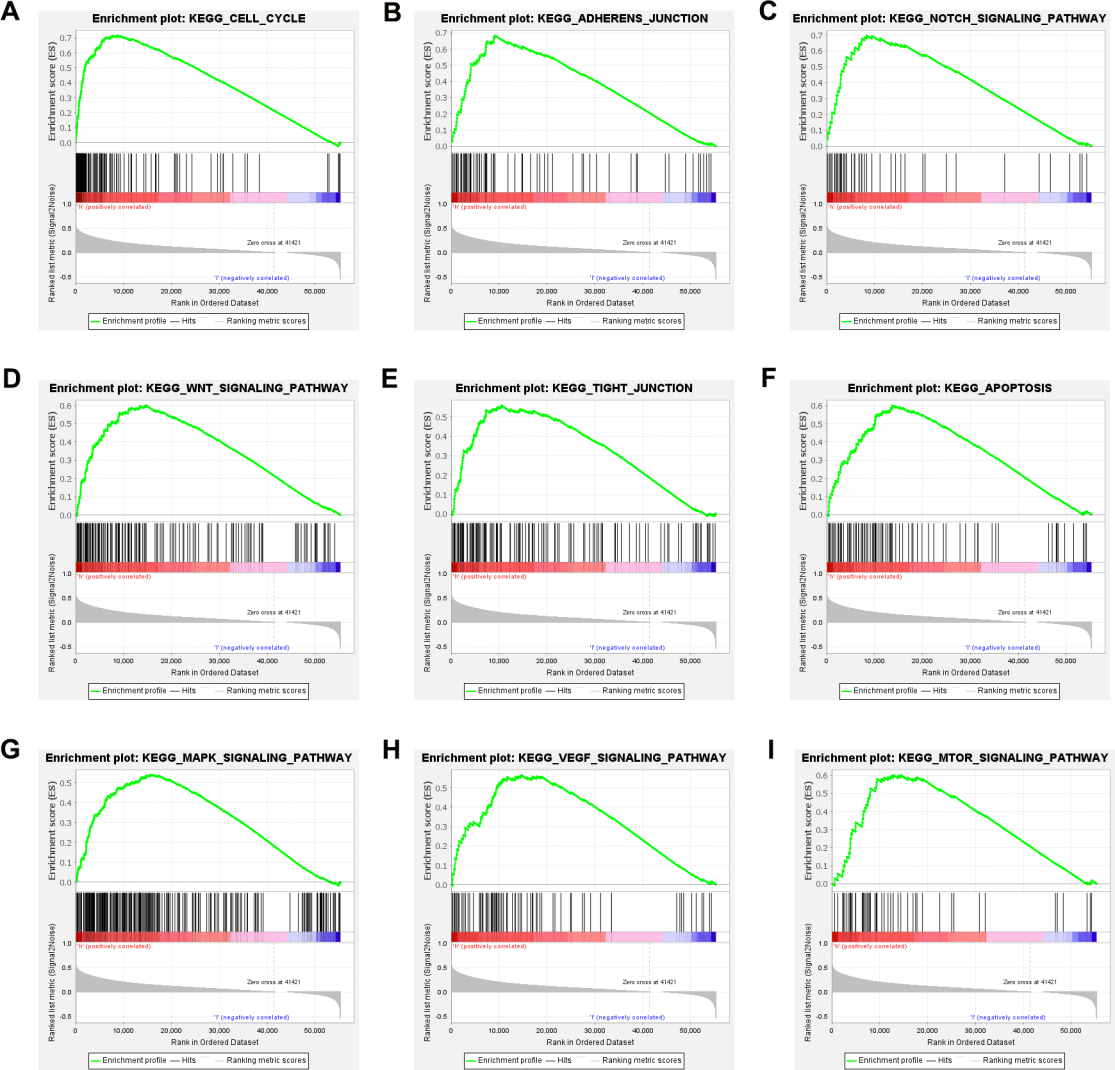
**High KIAA1522 expression is associated with cell cycle.** Enrichment plots from GSEA. GSEA results showing differential enrichment of genes related to cell cycle (**A**), adherens junction (**B**), tight junction (**C**), mTOR signaling pathway (**D**), Wnt signaling pathway (**E**), MAPK signaling pathway (**F**), beta signaling pathway (**G**), apoptosis (**H**) and p53 signaling pathway (**I**) in HCC cases with high KIAA1522 expression.

Gene sets related to cell cycle, adherens junction, tight junction, mTOR signaling pathway, Wnt signaling pathway, MAPK signaling pathway, beta signaling pathway, apoptosis and p53 signaling pathway were differentially enriched with the high KIAA1522 expression phenotype (Figure 5A–5I). GSEA revealed significant differences (NOM P-value <0.05 and FDR q-value <0.05) in the enrichment of Molecular Signatures Database (MSigDB) Collection, and the details were presented as Table 2. The NES related to cell cycle was the highest in high KIAA1522 expression of HCC cases.

**Table 2:** Gene sets enriched in phenotype high.

**Gene set name**	**NES**	**NOM p-Value**	**FDR q-Value**
KEGG_CELL_CYCLE	2.02	0.000	0.004
KEGG_ADHERENS_JUNCTION	2.00	0.000	0.004
KEGG_NOTCH_SIGNALING_PATHWAY	1.98	0.000	0.005
KEGG_WNT_SIGNALING_PATHWAY	1.98	0.000	0.004
KEGG_TIGHT_JUNCTION	1.92	0.000	0.009
KEGG_APOPTOSIS	1.88	0.000	0.009
KEGG_MAPK_SIGNALING_PATHWAY	1.88	0.000	0.009
KEGG_VEGF_SIGNALING_PATHWAY	1.87	0.000	0.009
KEGG_MTOR_SIGNALING_PATHWAY	1.83	0.008	0.011

NES: normalized enrichment score; NOM: nominal; FDR: false discovery rate. Gene sets with NOM P-value <0.05 and FDR q-value <0.05 were considered as significantly enriched.

### CYTOR affects proliferation, cell cycle and apoptosis of HCC cells by miR-125b-5p

**Figure 6:**
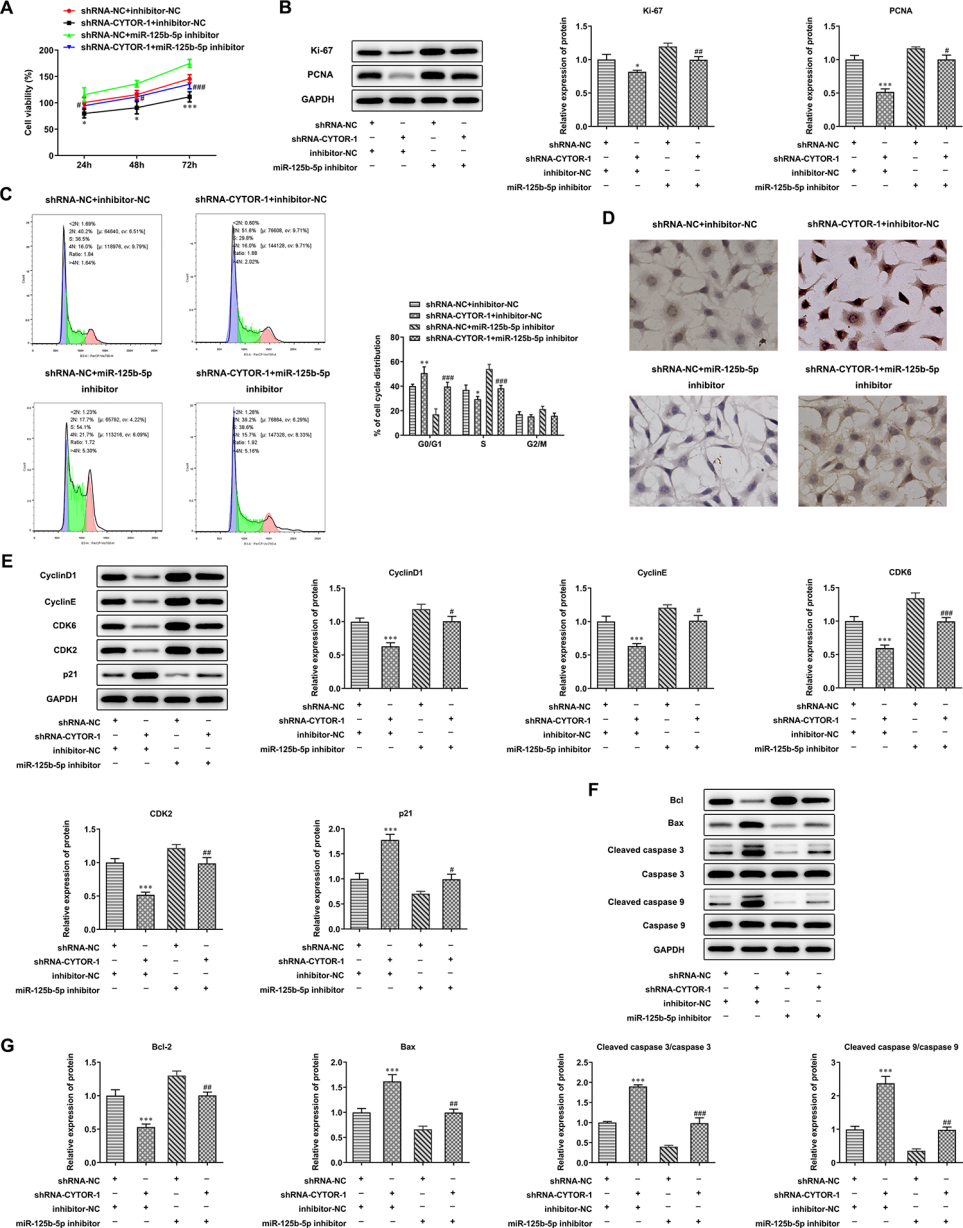
**CYTOR affects proliferation, cell cycle and apoptosis of HCC cells by miR-125b-5p.** (**A**) The proliferation of Hep3b cells after transfection of shRNA-CYTOR-1 and miR-125b-5p inhibitor was detected by CCK-8 assay. *P<0.05 and ***P<0.001 vs. shRNA-NC+inhibitor-NC group. ^#^P<0.05 and ^###^P<0.001 vs. shRNA-NC+miR-125b-5p inhibitor group. (**B**) The expression of proliferation-related proteins in Hep3b cells after transfection of shRNA-CYTOR-1 and miR-125b-5p inhibitor was detected by Western blot analysis. *P<0.05 and ***P<0.001 vs. shRNA-NC+inhibitor-NC group. ^#^P<0.05 and ^##^P<0.01 vs. shRNA-NC+miR-125b-5p inhibitor group. (**C**) The cell cycle of Hep3b cells after transfection of shRNA-CYTOR-1 and miR-125b-5p inhibitor was analyzed by flow cytometry analysis. *P<0.05 and **P<0.01 vs. shRNA-NC+inhibitor-NC group. ^###^P<0.001 vs. shRNA-NC+miR-125b-5p inhibitor group. (**D**) The apoptosis of Hep3b cells after transfection of shRNA-CYTOR-1 and miR-125b-5p inhibitor was shown by TUNEL assay. (**E**) The expression of cell cycle-related proteins in Hep3b cells after transfection of shRNA-CYTOR-1 and miR-125b-5p inhibitor was detected by Western blot analysis. ***P<0.001 vs. shRNA-NC+inhibitor-NC group. ^#^P<0.05, ^##^P<0.01 and ^###^P<0.001 vs. shRNA-NC+miR-125b-5p inhibitor group. (**F**, **G**) The expression of apoptosis-related proteins in Hep3b cells after transfection of shRNA-CYTOR-1 and miR-125b-5p inhibitor was detected by Western blot analysis. ***P<0.001 vs. shRNA-NC+inhibitor-NC group. ^##^P<0.01 and ^###^P<0.001 vs. shRNA-NC+miR-125b-5p inhibitor group.

As shown in Figure 6A, CYTOR interference suppressed the proliferation of Hep3b cells while miR- 125b-5p interference promoted the proliferation of Hep3b cells compared with that in the shRNA-NC + inhibitor-NC group. CYTOR interference decreased the expression of Ki-67 and PCNA in Hep3b cells, while their expression was increased by miR-125b-5p interference (Figure 6B). CYTOR interference increased the G0/G1 ratio and decreased the ratio of S phase and G2/M phase. Moreover, miR-125b-5p interference decreased the G0/G1 ratio and increased S ratio (Figure 6C). CYTOR interference down-regulated the expression of cyclinD1, CDK6, cyclinE and CDK2 and up-regulated p21 expression. Moreover, miR-125b-5p interference up-regulated the expression of cyclinD1, CDK6, cyclinE and CDK2, and down-regulated the p21 expression (Figure 6D). CYTOR interference promoted the apoptosis of Hep3b cells while miR-125b-5p interference inhibited the apoptosis of Hep3b cells (Figure 6E). CYTOR interference suppressed the Bcl-2 expression and promoted the expression of Bax, cleaved caspase 9 and cleaved caspase 3. Moreover, miR-125b-5p interference promoted the Bcl-2 expression and reduced the expression of Bax, cleaved caspase 9 and cleaved caspase 3 (Figure 6F, 6G). The effect of CYTOR interference on proliferation, cell cycle and apoptosis of Hep3b cells was alleviated by miR-125b-5p interference.

### MiR-125b-5p/KIAA1522 axis affects proliferation, cell cycle and apoptosis of HCC cells

**Figure 7:**
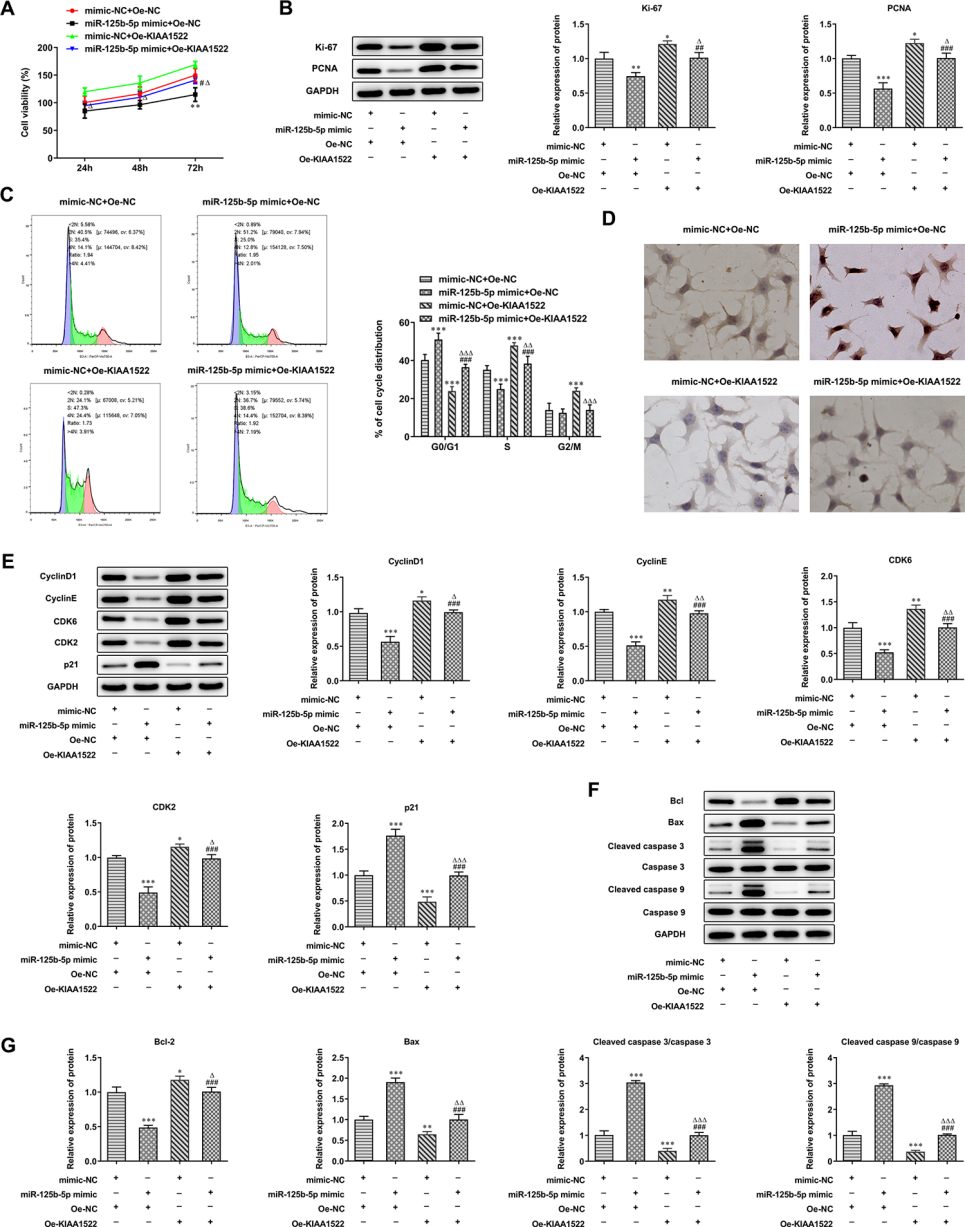
**MiR-125b-5p/KIAA1522 axis affects proliferation, cell cycle and apoptosis of HCC cells.** (**A**) The proliferation of Hep3b cells after transfection of miR-125b-5p mimic and Oe-KIAA1522 was detected by CCK-8 assay. **P<0.01 vs. mimic-NC+Oe-NC group. ^#^P<0.05 vs. miR-125b-5p mimic+Oe-NC group. ΔP<0.05 vs. mimic-NC+Oe-KIAA1522 group. (**B**) The expression of proliferation-related proteins in Hep3b cells after transfection of miR-125b-5p mimic and Oe-KIAA1522 was detected by Western blot analysis. *P<0.05, **P<0.01 and ***P<0.001vs. mimic-NC+Oe-NC group. ^##^P<0.01 and ^###^P<0.001 vs. miR-125b-5p mimic+Oe-NC group. ΔP<0.05 vs. mimic-NC+Oe-KIAA1522 group. (**C**) The cell cycle of Hep3b cells after transfection of miR-125b-5p mimic and Oe-KIAA1522 was analyzed by flow cytometry analysis. ***P<0.001vs. mimic-NC+Oe-NC group. ^###^P<0.001 vs. miR-125b-5p mimic+Oe-NC group. ΔΔP<0.01 and ΔΔΔP<0.001 vs. mimic-NC+Oe-KIAA1522 group. (**D**) The apoptosis of Hep3b cells after transfection of miR-125b-5p mimic and Oe-KIAA1522 was shown by TUNEL assay. (**E**) The expression of cell cycle-related proteins in Hep3b cells after transfection of miR-125b-5p mimic and Oe-KIAA1522 was detected by Western blot analysis. *P<0.05, **P<0.01 and ***P<0.001vs. mimic-NC+Oe-NC group. ^###^P<0.001 vs. miR-125b-5p mimic+Oe-NC group. ΔP<0.05, ΔΔP<0.01 and ΔΔΔP<0.001 vs. mimic-NC+Oe-KIAA1522 group. (**F**, **G**) The expression of apoptosis-related proteins in Hep3b cells after transfection of miR-125b-5p mimic and Oe-KIAA1522 was detected by Western blot analysis. *P<0.05, **P<0.01 and ***P<0.001vs. mimic-NC+Oe-NC group. ^###^P<0.001 vs. miR-125b-5p mimic+Oe-NC group. ΔP<0.05, ΔΔP<0.01 and ΔΔΔP<0.001 vs. mimic-NC+Oe-KIAA1522 group.

KIAA1522 expression was increased in Hep3b cells transfected with Oe-KIAA1522 compared with the control group and Oe-NC group (Figure 7A). MiR-125b-5p overexpression inhibited the proliferation of Hep3b cells and KIAA1522 overexpression promoted the proliferation of Hep3b cells (Figure 7B). MiR-125b-5p overexpression reduced the expression of Ki-67 and PCNA which was increased by KIAA1522 overexpression (Figure 7C). MiR-125b-5p overexpression increased the G0/G1 ratio and decreased the ratio of S phase and G2/M phase. Moreover, KIAA1522 overexpression decreased the G0/G1 ratio and increased S ratio (Figure 7D). MiR-125b-5p overexpression reduced the expression of cyclinD1, CDK6, cyclinE and CDK2 while promoted the p21 expression, which was reversed by KIAA1522 overexpression (Figure 7E). The apoptosis of Hep3b cells was increased by miR-125b-5p overexpression and decreased by KIAA1522 overexpression. MiR-125b-5p overexpression reduced the Bcl-2 expression and increased the expression of Bax, cleaved caspase 9 and cleaved caspase 3, which was reversed by KIAA1522 overexpression (Figure 7F, 7G). The effect of miR-125b-5p overexpression on proliferation, cell cycle and apoptosis of Hep3b cells was alleviated by KIAA1522 overexpression.

## DISCUSSION

Here, it was shown that the expression of CYTOR and KIAA1522 was increased and miR-125b-5p expression was decreased in HCC cells. High CYTOR expression was associated with high grade tumor. Kaplan-Meier curves for OS showed that higher expression of CYTOR and KIAA1522 was associated with worse outcomes in HCC patients. Univariate and multivariate Cox analyses indicated the CYTOR may be a useful biomarker for HCC prognosis.

In recent years, more and more studies have shown that lncRNA plays a key regulatory role in a variety of tumors [14, 15]. Like protein targets, lncRNA can also serve as an oncogene [16]. As a member of the lncRNA family, CYTOR was highly expressed in non-small cell lung cancer, gallbladder cancer and other malignant tumor tissues [17]. In non-small cell lung cancer, CYTOR promoted proliferation and invasion of tumor cells and increased resistance to radiation therapy [8]. In colorectal cancer cells, CYTOR promoted the proliferation of tumor cells and was considered to be an oncogene [18]. Zhu et al. found that down-regulation of CYTOR expression could negatively regulate the expression of miR-4775 and promote the proliferation and invasion of glioma cells [19]. Wang et al. demonstrated that down-regulation of CYTOR expression could inhibit the progression of gastric cancer by regulating the miR-193b-3p/ETS1 axis [20]. In this study, TCGA database indicated that CYTOR expression was increased in HCC tissues. High CYTOR expression was related to worse OS of HCC patients. CYTOR expression was the highest in HCC at stage IV, G4 and T4. In addition, CYTOR plays a similar role in HCC compared with previous research. CYTOR interference suppressed the proliferation and cell cycle, and promoted apoptosis of HCC cells.

MiRNAs are small non-coding RNAs (about 21-25 nucleotides in length) in cells. MiRNAs can negatively regulate the cell proliferation, differentiation and survival through protein-coding genes [21]. In recent years, several new studies have shown the abnormal expression of miR-125b-5p in many tumors, including laryngeal squamous cell carcinoma, esophageal squamous cell carcinoma, and multiple myeloma [9, 22, 23]. Morelli E et al. indicated that miR-125b-5p expression in multiple myeloma was decreased, which damaged the growth environment of multiple myeloma cells and decreased the survival rate of the cells [23]. Hua et al. demonstrated that miR-125b-5p expression was decreased in HCC tissues, and lower miR-125b-5p expression was related to the poor prognosis. Moreover, miR-125b-5p overexpression suppressed the proliferation, migration, and invasion of HCC cells [24]. The present study indicated that miR-125b-5p expression was also decreased in HCC cells. MiR-125b-5p expression was increased by CYTOR interference. MiR-125b-5p interference promoted proliferation and cell cycle, and inhibited apoptosis of HCC cells. The effect of CYTOR interference on HCC cells could be reserved by miR-125b-5p interference.

KIAA1522 is a newly cloned coding gene whose function is unknown [25]. Gene Expression Atlas and EMBL-EBI database information showed that KIAA1522 mRNA was up-regulated in lung cancer, breast cancer and other tumor tissues. Previous researches showed that KIAA1522 overexpression could promote the proliferation of human non-small cell lung cancer cells *in vitro*, suggesting that KIAA1522 could function as an oncogene [12, 26]. The present study demonstrated that KIAA1522 expression was increased in HCC cells and high KIAA1522 expression was related to cell cycle. KIAA1522 expression was decreased in HCC cells transfected with miR-125b-5p mimic and increased in HCC cells transfected with miR-125b-5p inhibitor. In addition, KIAA1522 overexpression promoted proliferation and cell cycle, and inhibited apoptosis of HCC cells, while these effects were reversed by miR-125b-5p overexpression. Above all, the roles of CYTOR, miR-125b-5p and KIAA1522 demonstrated in HCC were consistent with the finding of previous studies.

In conclusion, CYTOR and KIAA1522 were up-regulated while miR-125b-5p was down-regulated in HCC. CYTOR expression was related to clinical progression, and high CYTOR expression was an independent risk factor for worse OS in HCC patients. In addition, CYTOR interference suppressed proliferation and cell cycle, and promoted apoptosis of HCC cells by sponging miR-125b-5p that targeted KIAA1522. The present findings will provide a solid foundation for the prevention and treatment of HCC. However, some of limitations have existed in the present study. Due to the limitations *in vitro* experiment, the findings presented will need to be supported by further studies in animal experiments or in larger, independent patient cohorts.

## MATERIALS AND METHODS

### Bioinformatics analysis

Bioinformatics analysis was conducted using The Cancer Genome Atlas database (TCGA, https://cancergenome.nih.gov/). Gene expression RNAseq dataset of TCGA derived from HCC (50 normal and 374 cancer). The corresponding clinical information was also downloaded from TCGA. All data were processed using R software (version 3.5.1) [27].

### Cell culture

HHL-5 cells were provided from BioVector NTCC Inc. (Beijing, China). HCC cells (SK-Hep-1, Hep3b and Huh-7 cells) were obtained from the Cell Bank of Chinese Academy of Science (Shanghai, China). All cells were cultured in the 1640 medium (10% fetal bovine serum and 1% penicillin streptomycin) (Thermo fisher scientific, Shanghai, CN) at 37° C in a humidified atmosphere containing 5% CO_2_.

### Real-time quantitative polymerase chain reaction (RT-qPCR) analysis

TRIzol method was used to extract total RNA from cells and determine the concentration and purity of RNA (NanoDrop company, USA). In order to detect the expression of CYTOR, miR-125b-5p and KIAA1522 in cells and transfected cells, reverse transcription was performed according to the instructions attached to the reverse transcription test kit (Thermo fisher scientific, Shanghai, CN), and then quantitative detection was performed by ChamQ Universal SYBR PCR Master Mix (Beyotime, Shanghai, CN). U6 and GAPDH were used as internal reference. The real-time quantitative reaction was carried out on the ABI 7900 Fast Real Time PCR system. The 2^-ΔΔCt^ method was used to calculate the relative expression of CYTOR, miR-125b-5p and KIAA1522, and the experiment was repeated three times.

### Cell transfection

Hep3b cells in the logarithmic phase were cultured overnight in 6-well plates with a density of 3×10^5^/well. On the next day, shRNA-NC (20 nM), shRNA-CYTOR-1 (20 nM), shRNA-CYTOR-2 (20 nM), mimic-NC (50 nM), miR-125b-5p mimic (50 nM), inhibitor-NC (50 nM), miR-125b-5p inhibitor (50 nM), Oe-NC (50 nM), and Oe-KIAA1522 (50 nM) were respectively transfected into Hep3b cells with Lipofectamine® 2000 reagent according to the manufacturer’s protocol. Culture medium was changed 6 h after transfection, and cells were collected 36 h after transfection to verify the transfection efficiency for subsequent use. miR-125b-5p mimic sequence was 5′-UCCCUGAGACCCUAACUUGUCA-3′ and the miR-NC sequence was 5′- UUCUCCGACGUGUCACGUTT-3′. miR-125b-5p inhibitor sequence was 5′-UCACAAGUUAGGGUCUCAGGGA-3′ and the inhibitor-NC sequence was 5′-CAGUACUUUUGUGUAGUACAA-3′. The plasmids for shRNA-NC, shRNA-CYTOR-1/2, Oe-NC, and Oe-KIAA1522 were commercially synthesized by Shanghai GenePharma Co., Ltd.

### Patients and sample collection

The experimental design was approved by the Human Ethics Committee Review Board of Peking Union Medical College Hospital and informed consent was obtained from each patient. Paraffin-embedded specimens of HCC tissues and adjacent tissues were collected from 30 cases of untreated HCC patients. All paraffin-embedded specimens were provided from Pathology Department of Peking Union Medical College Hospital.

### Dual-luciferase reporter assay

To verify the prediction that CYTOR could combine with miR-125b-5p, Hep3b cells were co-transfected with CYTOR 3'UTR pmirGLO plasmid (containing WT CYTOR 3'UTR or MUT CYTOR 3'UTR) and a miR-125b-5p mimic or mimic-NC vector using Lipofectamine® 2000 reagent. To verify the prediction that miR-125b-5p could combine with KIAA1522, Hep3b cells were co-transfected with KIAA1522 3'UTR pmirGLO plasmid (containing WT KIAA1522 3'UTR or MUT KIAA1522 3'UTR) and a miR-125b-5p mimic or mimic-NC vector using Lipofectamine® 2000 reagent. Vector containing Renilla luciferase was used as control. The relative luciferase activity was detected by the Dual-Luciferase Reporter System (Promega Corporation, Madison, WI, USA).

### Immunohistochemistry

Paraformaldehyde (4%)-fixed and paraffin-embedded sections (4 μm) of HCC tissues and adjacent tissues were incubated with rabbit anti-KIAA1522 antibody (ab122203; Abcam). After washed with phosphate buffered saline (PBS), the sections were incubated with goat anti-rabbit IgG H&L (HRP) (ab205718; Abcam), followed by the treatment of 3,3′-diaminobenzidine chromogen substrate buffer. The sections were counterstained with hematoxylin and then mounted on slides. KIAA1522 expression was analyzed using Image J.

### Cell counting kit-8 (CCK-8) assay

The transfected Hep3b cells were prepared into 1×10^4^/mL cell suspensions. 100 μL cell suspension was added to each well in a 96-well plate, and wells only added with medium were considered as blank control. 10 μL CCK-8 solution (Beyotime, Shanghai, CN) was added to the corresponding wells at 0, 24, 48 and 72 h. After incubation for 2 h, the absorbance value at the wavelength of 450 nm was detected by an enzyme marker.

### Western blot analysis

After transfection, Hep3b cells were lysed with RIPA lysate containing 1% protease inhibitor and 1% phosphatase inhibitor (Beyotime, Shanghai, CN) to extract the total protein. After protein concentration was determined by BCA kit (Beyotime, Shanghai, CN), proteins were mixed with SDS-PAGE sample loading buffer in 100° C water bath for 5-10 min. After protein separation by SDS-PAGE electrophoresis, proteins with corresponding molecular weight were transferred to a PVDF membrane (Beyotime, Shanghai, CN). Then the membrane was sealed at room temperature with 50 g/L skim milk powder solution for 2 h. The primary antibodies containing Ki-67 (ab15580, Abcam), Proliferating cell nuclear antigen (PCNA) (ab18197, Abcam), cyclinD1 (ab40754, Abcam), CDK6 (ab124821, Abcam), cyclinE (ab33911, Abcam), Cyclin-dependent kinase 2 (CDK2) (ab32147, Abcam), p21 (ab109520, Abcam), B-cell lymphoma 2 (Bcl-2) (ab32124, Abcam), Bax (ab182733, Abcam), cleaved caspase 9 (ab2324, Abcam), cleaved caspase 3 (ab32042, Abcam), caspase 3 (ab13847, Abcam), and caspase 9 (ab32539, Abcam) were added to the membrane, which was incubated at 4° C overnight. After washing the membrane with TBST, the membrane was incubated with goat anti-rabbit IgG-HRP secondary antibody (ab205718, Abcam) at room temperature for 1 h. After Tris Buffered Saline Tween (TBST) washing, hypersensitive electrochemiluminescence (ECL) exposure solution (Beyotime, Shanghai, CN) was added to the membrane for analysis by gel imaging system.

### Flow cytometry analysis

After transfection, Hep3b cells were digested with trypsin without EDTA and centrifuged at 1 000 r/min for 5 min after digestion. Hep3b cells were washed with pre-cooling PBS twice and finally resuspended with 500 μL binding buffer in tubes. Each tube was added with 5 μL FITC Annexin and 5 μL PI (Beyotime, Shanghai, CN), and incubated at room temperature for 15 min in the dark. The cell cycle distribution was analyzed by flow cytometry within 1 h.

### TUNEL assay

The apoptosis of Hep3b cells was determined by TUNEL detection kit (Beyotime, Shanghai, CN). Briefly, the cell slides were digested by protease K, followed by the treatment of TdT and Biotin-dUTP. The cell slides were then sealed by the sealing liquid and treated with streptavidin-HRP working liquid and diaminobenzidine (DAB) color reagent successively. The apoptosis of Hep3b cells color was observed by a light microscope.

### Gene set enrichment analysis (GSEA)

To identify potential mechanisms underlying the effect of KIAA1522 expression on HCC, GSEA was performed to identify gene sets and pathways correlated with high KIAA1522 expression. The pathways enriched in each phenotype was sorted by the nominal p value (NOM p-value) and normalized enrichment score (NES).

### Statistical analysis

All statistical analyses were conducted using R (version 3.5.1). The relationship between overall survival (OS) and CYTOR expression was analyzed by Kaplan-Meier survival analysis. The relationship between clinical pathological characteristics and CYTOR expression was analyzed with the Wilcoxon signed-rank test. Cox regression analysis was used to analyze the relationship of CYTOR expression with other clinical characteristics. All experimental data were reported as mean ± standard deviation. Multiple comparisons in different groups were evaluated by the one-way ANOVA. P<0.05 was considered statistically significant.

### Ethics approval

The experimental design was approved by the Human Ethics Committee Review Board of Peking Union Medical College Hospital and informed consent was obtained from each patient.

### Availability of data and material

Data will be available on the request.

## References

[1] Bray F, Ferlay J, Soerjomataram I, Siegel RL, Torre LA, Jemal A. Global cancer statistics 2018: GLOBOCAN estimates of incidence and mortality worldwide for 36 cancers in 185 countries. CA Cancer J Clin. 2018; 68:394–424. 10.3322/caac.21492 30207593

[2] Villanueva A. Hepatocellular carcinoma. N Engl J Med. 2019; 380:1450–62. 10.1056/NEJMra1713263 30970190

[3] Chen W, Xia C, Zheng R, Zhou M, Lin C, Zeng H, Zhang S, Wang L, Yang Z, Sun K, Li H, Brown MD, Islami F, et al. Disparities by province, age, and sex in site-specific cancer burden attributable to 23 potentially modifiable risk factors in China: a comparative risk assessment. Lancet Glob Health. 2019; 7:e257–69. 10.1016/S2214-109X(18)30488-1 30683243

[4] Thomas MB, Zhu AX. Hepatocellular carcinoma: the need for progress. J Clin Oncol. 2005; 23:2892–99. 10.1200/JCO.2005.03.196 15860847

[5] Spizzo R, Almeida MI, Colombatti A, Calin GA. Long non-coding RNAs and cancer: a new frontier of translational research? Oncogene. 2012; 31:4577–87. 10.1038/onc.2011.621 22266873 PMC3433647

[6] Zou SF, Yang XY, Li JB, Ding H, Bao YY, Xu J. UPF1 alleviates the progression of glioma via targeting lncRNA CYTOR. Eur Rev Med Pharmacol Sci. 2019; 23:10005–12. 10.26355/eurrev_201911_19567 31799670

[7] Li M, Wang Q, Xue F, Wu Y. lncRNA- CYTOR works as an oncogene through the CYTOR/miR-3679-5p/ MACC1 axis in colorectal cancer. DNA Cell Biol. 2019; 38:572–82. 10.1089/dna.2018.4548 31144988

[8] Zhang J, Li W. Long noncoding RNA CYTOR sponges miR-195 to modulate proliferation, migration, invasion and radiosensitivity in nonsmall cell lung cancer cells. Biosci Rep. 2018; 38:BSR20181599. 10.1042/BSR20181599 30487160 PMC6435535

[9] Li Y, Wang Y, Fan H, Zhang Z, Li N. miR-125b-5p inhibits breast cancer cell proliferation, migration and invasion by targeting KIAA1522. Biochem Biophys Res Commun. 2018; 504:277–82. 10.1016/j.bbrc.2018.08.172 30177391

[10] Mei LL, Wang WJ, Qiu YT, Xie XF, Bai J, Shi ZZ. miR-125b-5p functions as a tumor suppressor gene partially by regulating HMGA2 in esophageal squamous cell carcinoma. PLoS One. 2017; 12:e0185636. 10.1371/journal.pone.0185636 28968424 PMC5624607

[11] Liu S, Chen Q, Wang Y. MiR-125b-5p suppresses the bladder cancer progression via targeting HK2 and suppressing PI3K/AKT pathway. Hum Cell. 2020; 33:185–94. 10.1007/s13577-019-00285-x 31605287

[12] Liu YZ, Yang H, Cao J, Jiang YY, Hao JJ, Xu X, Cai Y, Wang MR. KIAA1522 is a novel prognostic biomarker in patients with non-small cell lung cancer. Sci Rep. 2016; 6:24786. 10.1038/srep24786 27098511 PMC4838871

[13] Xie ZH, Yu J, Shang L, Zhu YQ, Hao JJ, Cai Y, Xu X, Zhang Y, Wang MR. KIAA1522 overexpression promotes tumorigenicity and metastasis of esophageal cancer cells through potentiating the ERK activity. Onco Targets Ther. 2017; 10:3743–54. 10.2147/OTT.S142610 28794639 PMC5538704

[14] Bhan A, Soleimani M, Mandal SS. Long noncoding RNA and cancer: a new paradigm. Cancer Res. 2017; 77:3965–81. 10.1158/0008-5472.CAN-16-2634 28701486 PMC8330958

[15] Li J, Li Z, Zheng W, Li X, Wang Z, Cui Y, Jiang X. LncRNA-ATB: an indispensable cancer-related long noncoding RNA. Cell Prolif. 2017; 50:e12381. 10.1111/cpr.12381 28884871 PMC6529097

[16] Peng WX, Koirala P, Mo YY. LncRNA-mediated regulation of cell signaling in cancer. Oncogene. 2017; 36:5661–67. 10.1038/onc.2017.184 28604750 PMC6450570

[17] Liang J, Wei X, Liu Z, Cao D, Tang Y, Zou Z, Zhou C, Lu Y. Long noncoding RNA CYTOR in cancer: a TCGA data review. Clin Chim Acta. 2018; 483:227–33. 10.1016/j.cca.2018.05.010 29750962

[18] Wang X, Yu H, Sun W, Kong J, Zhang L, Tang J, Wang J, Xu E, Lai M, Zhang H. The long non-coding RNA CYTOR drives colorectal cancer progression by interacting with NCL and Sam68. Mol Cancer. 2018; 17:110. 10.1186/s12943-018-0860-7 30064438 PMC6069835

[19] Zhu Z, Dai J, Liao Y, Ma J, Zhou W. Knockdown of long noncoding RNA LINC00152 suppresses cellular proliferation and invasion in glioma cells by regulating miR-4775. Oncol Res. 2018; 26:857–67. 10.3727/096504017X15016337254597 28800786 PMC7844725

[20] Wang H, Chen W, Yang P, Zhou J, Wang K, Tao Q. Knockdown of linc00152 inhibits the progression of gastric cancer by regulating microRNA-193b-3p/ETS1 axis. Cancer Biol Ther. 2019; 20:461–73. 10.1080/15384047.2018.1529124 30404587 PMC6422511

[21] Hayes J, Peruzzi PP, Lawler S. MicroRNAs in cancer: biomarkers, functions and therapy. Trends Mol Med. 2014; 20:460–69. 10.1016/j.molmed.2014.06.005 25027972

[22] Hui L, Zhang J, Guo X. MiR-125b-5p suppressed the glycolysis of laryngeal squamous cell carcinoma by down-regulating hexokinase-2. Biomed Pharmacother. 2018; 103:1194–201. 10.1016/j.biopha.2018.04.098 29864898

[23] Morelli E, Leone E, Cantafio ME, Di Martino MT, Amodio N, Biamonte L, Gullà A, Foresta U, Pitari MR, Botta C, Rossi M, Neri A, Munshi NC, et al. Selective targeting of IRF4 by synthetic microRNA-125b-5p mimics induces anti-multiple myeloma activity *in vitro* and *in vivo*. Leukemia. 2015; 29:2173–83. 10.1038/leu.2015.124 25987254 PMC4635336

[24] Hua S, Quan Y, Zhan M, Liao H, Li Y, Lu L. miR-125b-5p inhibits cell proliferation, migration, and invasion in hepatocellular carcinoma via targeting TXNRD1. Cancer Cell Int. 2019; 19:203. 10.1186/s12935-019-0919-6 31384178 PMC6668076

[25] Nagase T, Kikuno R, Hattori A, Kondo Y, Okumura K, Ohara O. Prediction of the coding sequences of unidentified human genes. Xix. The complete sequences of 100 new cDNA clones from brain which code for large proteins *in vitro*. DNA Res. 2000; 7:347–55. 10.1093/dnares/7.6.347 11214970

[26] Liu YZ, Jiang YY, Wang BS, Hao JJ, Shang L, Zhang TT, Cao J, Xu X, Zhan QM, Wang MR. A panel of protein markers for the early detection of lung cancer with bronchial brushing specimens. Cancer Cytopathol. 2014; 122:833–41. 10.1002/cncy.21461 25045014

[27] Gentleman R, Ihaka R. A language and environment for statistical computing. Computing. 2011; 1:12–21.

